# Severity‐dependent recovery time in acute lateral ankle sprains: An ultrasonographic assessment of talofibular displacement

**DOI:** 10.1002/jeo2.70204

**Published:** 2025-03-22

**Authors:** Yuto Uchida, Masashi Kawabata, Yusuke Kumazawa, Kazuya Takagi, Kazuma Miyatake, Takumi Kobayashi, Tomonori Kenmoku, Hiroyuki Watanabe, Naonobu Takahira

**Affiliations:** ^1^ Department of Sports Medicine Kitasato University Graduate School of Medical Sciences Sagamihara Japan; ^2^ Rehabilitation Center Sagamihara Kyodo Hospital Sagamihara Japan; ^3^ Department of Rehabilitation Kitasato University School of Allied Health Sciences Sagamihara Japan; ^4^ Kumazawa Orthopaedic Clinic Tokyo Japan; ^5^ Konica Minolta, Inc. Tokyo Japan; ^6^ Department of Orthopaedic Surgery Yokohama City University Yokohama Japan; ^7^ Department of Rehabilitation Sciences Graduate School of Health Sciences Gunma University Maebashi Japan; ^8^ Department of Orthopaedic Surgery Kitasato University School of Medicine Sagamihara Japan

**Keywords:** ankle sprain, ankle injuries, joint laxity, ultrasonography, ultrasound imaging

## Abstract

**Purpose:**

There is no consensus on treatment protocols based on severity and timing for acute lateral ankle sprain (LAS). Appropriate decision‐making is necessary to prevent reinjury or chronic ankle instability. In this retrospective observational study, we clarified the duration of recovery from anterior ankle joint displacement in patients with initial acute LAS of several severities.

**Methods:**

Overall, 101 patients with varying grades of initial unilateral LAS were included. Injury severity was based on ligament tears and anterior ankle joint displacement using the reverse anterior drawer test with ultrasonography. The automated length measurement system software measured changes in the talofibular distance.

**Results:**

The median (95% confidence interval) change in the talofibular distance on the affected side was 1.24 (0.96–1.76), 3.03 (2.91–3.74) and 3.06 (2.37–4.69) mm for LAS grades I, II and III, respectively, on the first medical examination. The increase in talofibular distance for grade I injuries was significantly smaller than for Grades II and III (*p* < 0.01). The regression equation was *y* = –0.02 × days + 1.43, –0.05 × days + 3.30 and –0.05 × days + 3.42 for Grades I, II and III, respectively; the time it took to reach the value of the unaffected side was 14.5, 43.2 and 45.6 days, respectively. Regression coefficients were significantly greater for Grades II and III than for Grade I (*p* < 0.01 and *p* = 0.01, respectively). No significant differences were observed between Grades II and III.

**Conclusion:**

These results revealed that the recovery time for displacement varies according to the severity of the sprain, suggesting the need to develop optimal treatment protocols.

**Level of Evidence:**

Level III.

**Abbreviations**
ALMS: automated length measurement system
ATFL: anterior talofibular ligament
CAI: chronic ankle instability
LAS: lateral ankle sprain
R‐ADT: reverse anterior drawer test
ROI: region of interest
RTS: return to sport
US: ultrasound

## INTRODUCTION

Lateral ankle sprain (LAS) is the most frequently occurring sport injury, and > 50% of patients are reinjured [15]. Although most LASs heal with conservative treatment and allow the patient to return to sports (RTS), 40% of patients with initial LAS develop chronic ankle instability (CAI) [5]. CAI results in functional deficits, such as ankle dorsiflexion limitation and plantar flexion weakness, including proprioceptive deficits, leading to poor athletic performance [15, 24]. Furthermore, it increases the risk of ankle osteoarthritis as a long‐term prognosis [15, 16].

Wikstrom et al. [26] reported that an appropriate rest period after LAS could prevent adverse effects on joint structure and movement patterns leading to CAI. Hubbard and Cordova [8] also reported that anterior ankle joint displacement persisted after 8 weeks in patients with a first unilateral injury without a rest period. Despite the need for an adequate rest period after LAS, more than half of patients with CAI remain untreated after their first LAS [10], suggesting that they don't receive an adequate rest period after injury [23]. In addition, epidemiological studies in the United States have reported a median time to RTS after an initial LAS injury of 3 days, with approximately 90% of injured patients returning within 1 week [3, 18, 20, 21]. Early in the post‐injury period, when ligament healing is inadequate, joint displacement nor ankle joint function is likely fully restored for RTS [1, 4, 6]. Allowing high‐intensity activity in such situations may increase reinjury rates and contribute to higher transition rates to CAI.

Appropriate measures must be taken at the time of the first injury to prevent recurrence or severe injury after LAS, depending on its severity. However, no consensus has yet been reached regarding the treatment plan or rest period according to the severity of LAS [25]. Disease severity in LAS is generally classified into three grades [12]. Grade I involves ligament stretching without macroscopic tearing. Grade II is characterised by a partial macroscopic ligament tear. Grade III involves a completely ruptured ligament. The joint experiences abnormal motion and displacement, leading to functional loss [12]. This evaluation is objective and excludes any subjective assessment [12].

The ultrasound (US)‐guided anterior ankle joint stress test visualises ligament tears and joint displacement, enabling early diagnosis of injury severity [22]. However, no studies have quantified the time required to improve anterior ankle joint displacement based on severity. Determining this period would foster a common understanding among healthcare professionals and patients, potentially reducing the reinjury rate. Therefore, we aimed to quantitatively evaluate anterior ankle joint displacement over time in patients with initial acute LAS and to determine the time required to improve anterior ankle joint displacement in each of the three severity grades. It was hypothesised that the recovery duration would be longer with increasing severity.

## METHODS

### Study design and ethical considerations

This was a retrospective observational study based on existing medical data. The study posted an opt‐out notice in the hospital and guaranteed the right to refuse participation. As only anonymized clinical data were used for analysis, the requirement for informed consent was waived by the committee prior to the initiation of this retrospective study.

This study was conducted in accordance with the Declaration of Helsinki and was approved by the Ethics Committee of Kitasato University (study number 2021‐025‐2).

### Participants

This study included patients who underwent clinical examination from April 1, 2021 to March 31, 2023, and were diagnosed with LAS after excluding fracture cases using simple radiographic and US examinations. Out of 141 recruited patients, 101 (13, 78 and 10 patients with Grade I, II and III injuries, respectively) were selected for analysis. The median number (interquartile range) of days from the date of injury to the date of first medical examination was 1 (1–2) day (Table 1).

**Table 1:** Patient characteristics.

	Grade I (*n* = 13)	Grade II (*n* = 78)	Grade III (*n* = 10)	Overall (*n* = 101)
Sex, male (%)	6 (46.2)	51 (65.4)	4 (40.0)	61 (60.4)
Age (years)	13 [13–15.5]	16 [15–20]	17 [14–21]	16 [15–20]
Injured side, right (%)	2 (15.4)	40 (51.3)	5 (50.0)	47 (46.5)
Combined injury with the CFL (%)	0 (0)	10 (12.8)	0 (0)	10 (10)
Combined injury with the AITFL (%)	0 (0)	0 (0)	0 (0)	0 (0)
Number of days from the date of injury to the date of first medical examination (days)	1 [1–2]	2 [1–2.5]	1 [0.75–2]	1 [1–2]

*Note*: Median [interquartile range].

Abbreviations: AITFL, anterior inferior talofibular ligament; CFL, calcaneofibular ligament.

**Figure 1:**
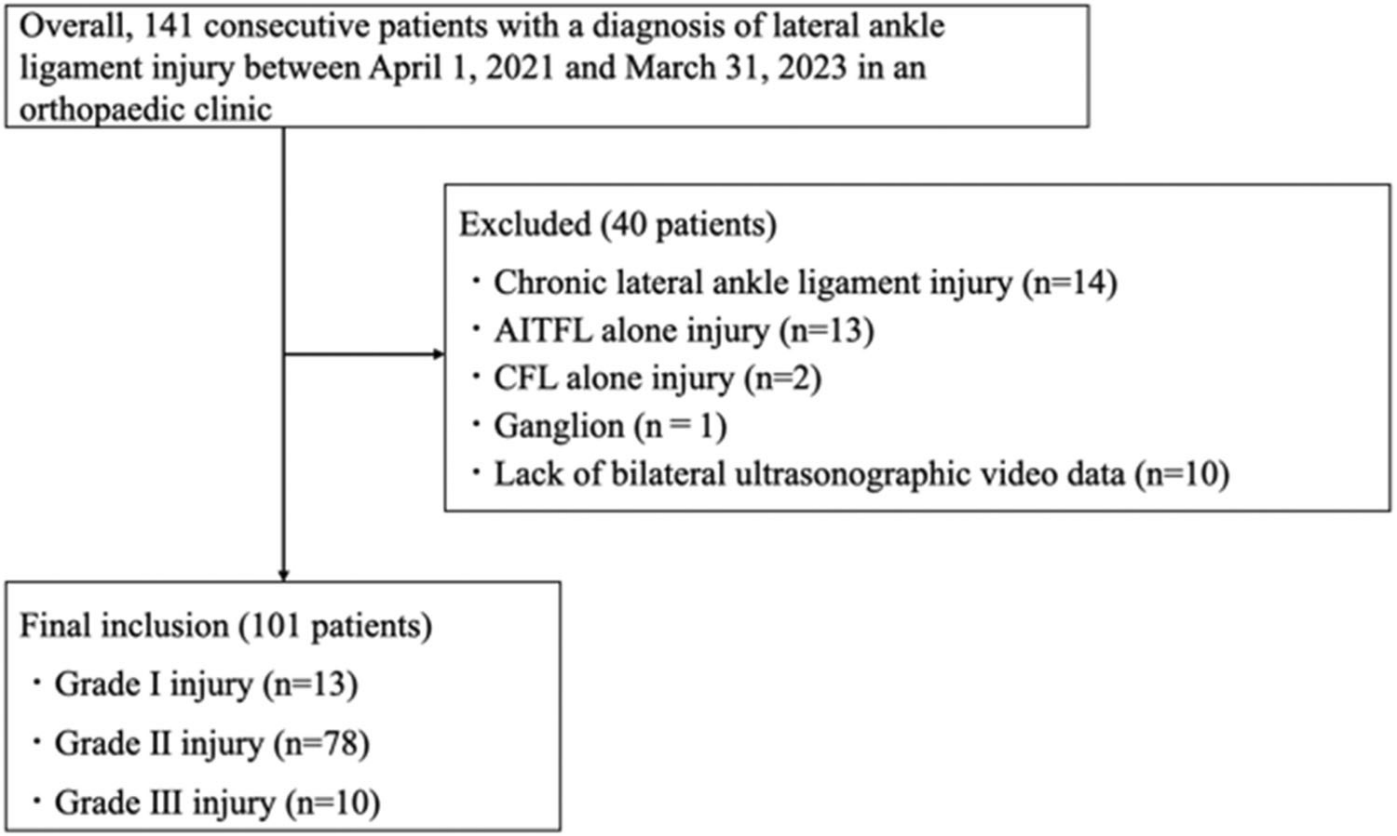
Flowchart of the participant selection process. AITFL, anterior inferior talofibular ligament; CFL, calcaneofibular ligament.

The inclusion criteria were as follows: first injury to the patients' feet, unilateral injury, injury to the anterior talofibular ligament (ATFL) alone, or combined injury to the ATFL with the calcaneofibular ligament or anterior inferior tibiofibular ligament. The number of times wherein the patients' feet were injured was determined using a questionnaire administered at the first medical examination. The exclusion criteria are presented in Figure 1.

### Clinical flowchart by severity of illness

#### Flowchart for diagnosis and severity determination of LAS

**Figure 2:**
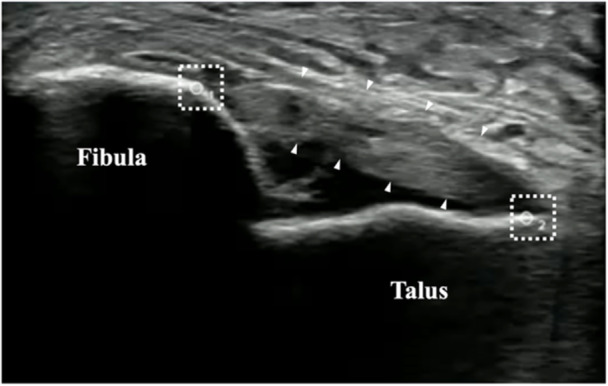
Ultrasonographic video imaging sites. ▲ anterior talofibular ligament (ATFL). 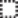 Region of interest (ROI).

The diagnosis and severity of LAS were based on the following procedures: after a simple radiographic examination of the ankle joint without fracture, the site and severity of ligament damage were assessed using a US imaging system. US evaluation was conducted on all patients by the same examiner, who had 5 years of US experience. Anterior ankle joint displacement was evaluated using the US‐guided reverse anterior drawer test (R‐ADT). R‐ADT was conducted as described by Iwata et al. [11]. Stress intensity was applied up to the endpoint while observing the ATFL under US; [11] however, stress could not be applied up to the endpoint in some patients due to pain and swelling immediately after the injury. The examiner used an ultrasonographic probe (L18‐4 Linear Array Transducer; Sonimage HS1 SNiBLE; Konica Minolta, Inc., Tokyo, Japan) coated with US gel to delineate the ATFL long‐axis images of the anterolateral ankle joint and ligament attachment areas (the lateral malleolus of the fibula and anterolateral talar body) (Figure 2).

An orthopaedic surgeon diagnosed severity based on the dynamics of the joint and the condition of the ligament tear area during R‐ADT at the first medical examination. As reported by Kannus et al., the severity of injury could be graded as follows: Grade I for a stretched ligament with mild swelling and tenderness, Grade II for a partial tear with moderate joint displacement and Grade III for a complete tear with prominent joint displacement [12].

During regular follow‐up examinations, R‐ADT was performed on the affected side to assess joint displacement over time and to aid in determining the treatment plan. The follow‐up frequency was set as follows: Grade I, voluntary; Grade II, at 1 week and 3 weeks, then every 2 weeks thereafter; and Grade III, at 1 week, 2 weeks, and 3 weeks, then every 2 weeks thereafter for a maximum of 60 days.

#### Treatment protocols for the severity of LAS

**Figure 3:**
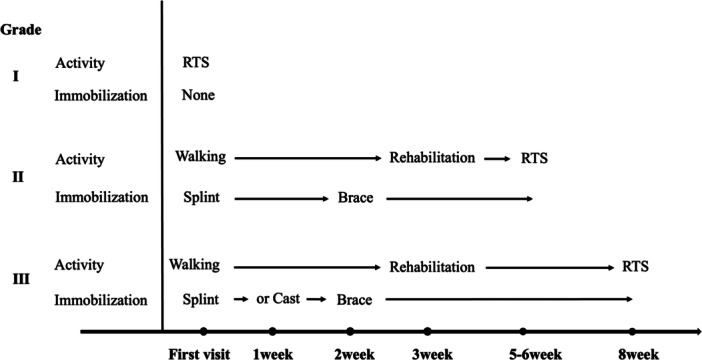
Protocol for the frequency of consultations and RTS for patients with LAS. LAS, lateral ankle sprain; RTS, return to sport.

Our clinic has established a protocol for the frequency of consultations and RTS after the first medical examination with LAS (Figure 3).

Grade I did not dictate periodic follow‐up examinations after the first medical examination and allowed for RTS as soon as muscle strength and joint range of motion are restored, based on educational guidance on self‐exercise. Grade II was monitored at 1 and 3 weeks then, every 2 weeks until RTS. Patients were instructed to use an L‐shaped immobilisation device for 7–10 days, followed by a functional brace (WRAPTOR; Shilac Japan Co., Ltd. Osaka, Japan) for up to 6 weeks. No weight restrictions were imposed initially, allowing patients to walk as early as possible. Patients experiencing pain and weight‐bearing difficulty were advised to use crutches to reduce the load. High‐intensity exercises, such as jogging, sprinting, jumping, and cutting manoeuvres, which are more intense than walking, were gradually reintroduced after approximately 3 weeks of active functional training with a physiotherapist. RTS was targeted at 5–6 weeks, with the initiation of high‐intensity exercises tailored to individual conditions and supervised by the attending physiotherapist. Grade III injuries followed a similar protocol to Grade II injuries, with additional follow‐ups at 2 weeks. External fixation was maintained using an L‐shaped splint for 2 weeks post‐assessment. For patients with significant anterior ankle joint displacement and loading difficulties, cast fixation was maintained for up to 2 weeks. The functional brace was used for 2–8 weeks, aiming for RTS at approximately 8 weeks.

#### Measurement of the distance between the talus and fibula (change in the talofibular distance)

The ultrasonographic video recorded during R‐ADT was saved on an external hard disk and imported to an offline personal computer. One trial of R‐ADT with clearly delineated bone contours and ATFL was cropped for data analysis using video editing software (Filmora, Wondershare Technology Co., Shenzhen, China) on an offline personal computer. The decision regarding the trimmed interval was made after consultation between the two analysts. An automated length measurement system (ALMS; JP‐A 2022‐66490; Konica Minolta, Inc., Tokyo, Japan) software was used to measure the distance between the two points on the talus and fibula. The ALMS software automatically followed the movement of the region of interest (ROI) in the bone contour and measured the distance between two points. The measurement error of this ALMS software is <0.4 mm, making it a measurement method with excellent validity and reproducibility [14]. Following the method described by Kawabata et al. [14], the same analyst manually placed the ROI on the bony contours of the talus and fibula (apex of the high‐echoic area) (Figure 2). The change in the talofibular distance was calculated from the minimum and maximum values of the two ROIs in an ultrasonographic video (30 Hz). The reproducibility of the measurement for the change in the talofibular distance was assessed through a preliminary test‐retest analysis of 20 randomly selected data points, which demonstrated excellent reliability, with an ICC (2,1) of 0.91. This result indicates a high level of consistency in image‐based distance measurements across repeated evaluations. Recovery was defined by achieving equivalence with the unaffected side as the criterion.

### Statistical analysis

A priori power analysis using G*Power software (version 3.1.9.2; University of Kiel, Kiel, Germany) indicated that, with a significance level of 0.05, a sample size of more than 55 participants would be required to achieve 80% power for detecting a moderate effect size. Age and number of days from the date of injury to the date of the first medical examination are shown as medians (interquartile ranges), whereas sex and site of injury are presented as frequencies. Continuous data were subjected to the Shapiro–Wilk test to confirm the normality of the data. The difference in the change in the talofibular distance between the affected and unaffected sides was compared using the Wilcoxon signed‐rank sum test, and those of severities were compared using the Kruskal–Wallis test, including the Steel–Dwass method for multiple comparisons. In addition, a single regression analysis was performed using a mixed model to calculate the number of days until the predicted value of the regression equation was the median change in the talofibular distance at the first medical examination for the unaffected side. For subgroup analysis, the regression coefficients of severity were compared using an F‐test with a mixed model. All statistical analyses were performed using JMP® Pro 17 (JMP Statistical Discovery LLC., Cary, NC, USA), with a significance level of <5% risk.

## RESULTS

### Amount of change in the talofibular distance on the date of the first medical examination

**Figure 4:**
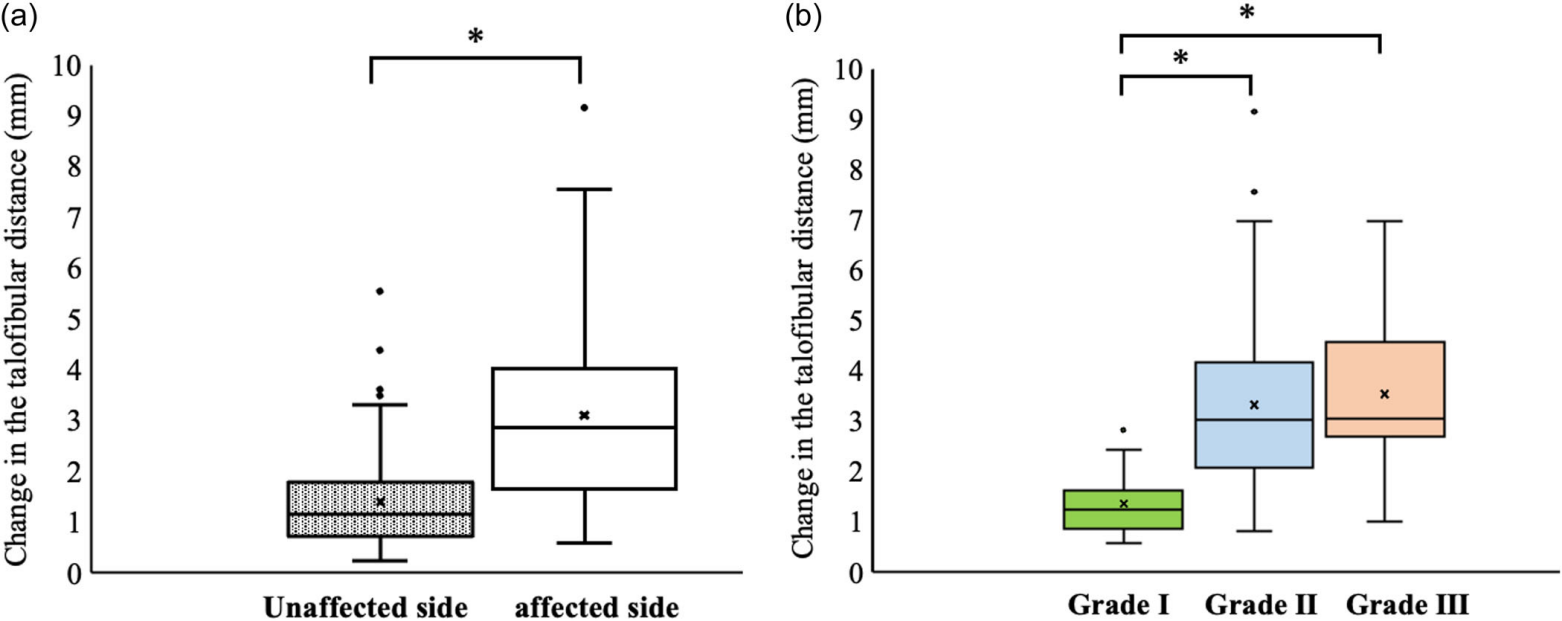
Change in the talofibular distance between the affected and unaffected sides at the first medical examination in overall patients (a) and on the affected side by severity (b).

Patient characteristics are summarised in Table 1. The median (95% confidence interval [CI]) change in the talofibular distance at the first medical examination was 2.86 (2.75–3.47) mm for the affected side and 1.14 (1.20–1.57) mm for the unaffected side, showing a significant difference (*p* < 0.01) (Figure 4a).

Furthermore, the median (95% CI) change in the talofibular distance on the affected side at the first medical examination was 1.24 (0.96–1.76), 3.03 (2.91–3.74) and 3.06 (2.37–4.69) mm for Grade I, II and III injuries, respectively. The change in talofibular distance for Grade I injuries was significantly smaller than for Grades II and III (all *p* < 0.01); however, no significant difference was observed between Grades II and III (*ns*) (Figure 4b).

### Amount of change in the talofibular distance on the affected side with respect to the number of days

**Figure 5:**
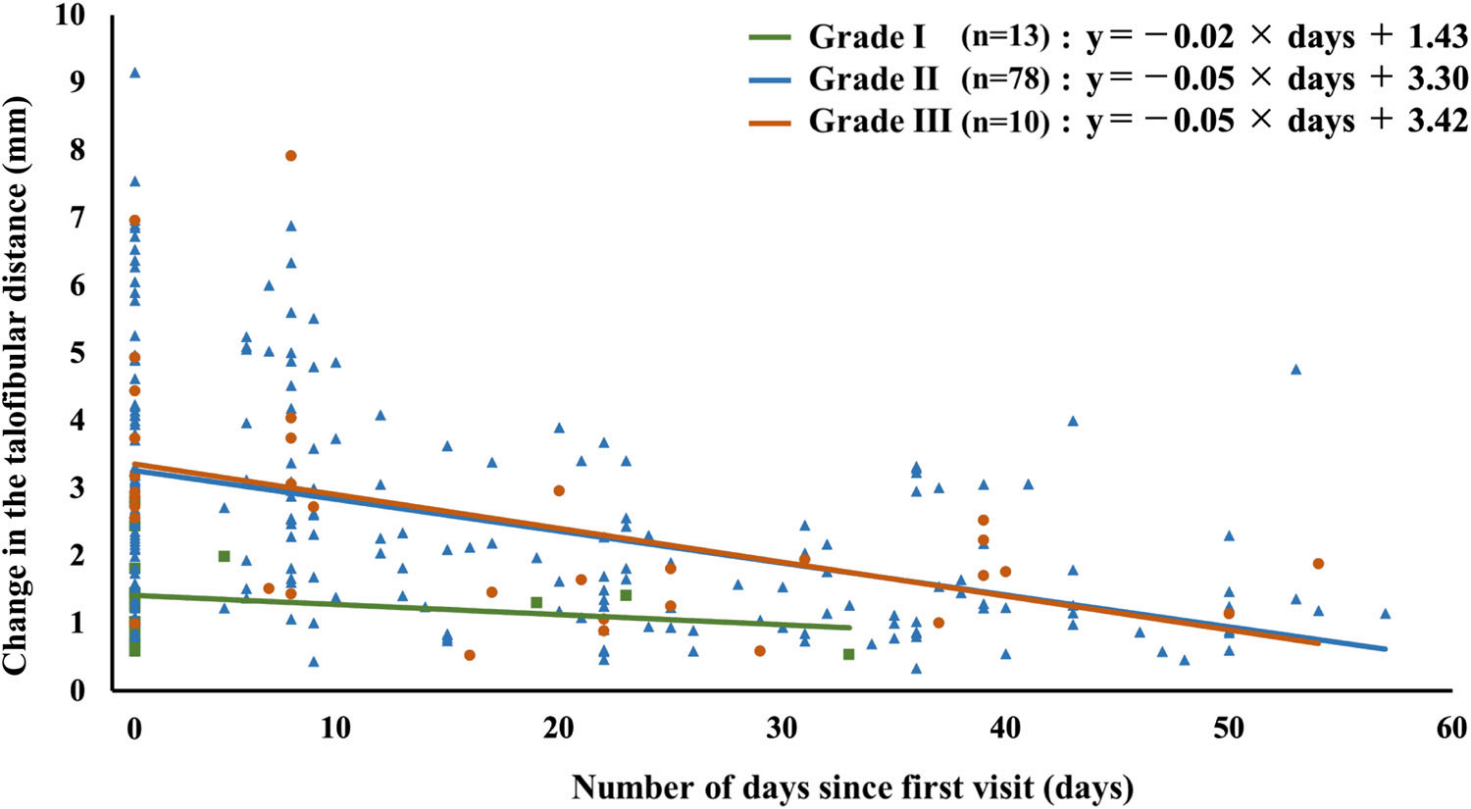
Amount of change in the talofibular distance and the regression equation on the affected side relative to the number of days. ■ Grade I, ▲ Grade II, ● Grade III.

The regression equation was calculated from 276 observations of patients who met the inclusion criteria. The regression equation by severity is shown in Figure 5. Regression coefficients by severity were significantly greater for grades II and III compared to the corresponding coefficients for Grade I (*p* < 0.01 and *p* = 0.01, respectively). No significant difference was observed between Grades II and III (*ns*). In the regression equation, the estimated duration for reaching the level of the unaffected side was 14.5, 43.2 and 45.6 days for Grades I, II and III, respectively.

## DISCUSSION

The most important finding of the present study was that the estimated time to recover anterior ankle joint displacement in patients with initial acute LAS differed between LAS Grades I and II–III, but not between Grades II and III. This is the first report using US‐guided anterior drawer test to track changes in anterior ankle joint displacement over time in initial acute LAS. The findings of this study are expected to contribute to a common understanding among healthcare providers and patients, aiding in decision‐making regarding the appropriate rest duration for initial acute LAS.

### The change in the talofibular distance on the date of the first medical examination

In the present study, the change in the talofibular distance at the first medical examination was significantly greater on the affected side compared to that in the unaffected side (Figure 4a). Hubbard and Cordova [8] also reported greater anterior ankle joint displacement on the affected side at 3 days post‐injury in the initial acute LAS. When classified by severity, the change in the talofibular distance on the affected side was significantly smaller for Grade I injuries than for Grade II and III injuries. However, contrary to our hypothesis, there was no difference between Grade II and III injuries (Figure 4b). Similar findings were reported by Gulick [7] using the Mobil‐Aider™ device (Therapeutic Articulations, LLC, Spring City, PA, USA). Furthermore, it was noted by Wisthoff et al. [27] that measurements using US with a Telos Stress device (Telos Health, Campbell, CA, USA) showed smaller changes in Grade I injuries compared to Grades II and III within 24–72 h after LAS. While Grade I had significantly lesser displacement than Grades II and III, no difference was observed between Grades II and III. This study did not quantify pain, inflammation, and swelling, but joint mobility was smaller in some Grades II and III cases due to these symptoms. As these symptoms reduced, joint mobility increased in some cases during the second examination compared to the first. This may explain the larger 95% CI for Grades II (2.91–3.74 mm) and III (2.37–4.69 mm) compared to Grade I (0.96–1.76 mm). Therefore, determining the severity of acute LAS is crucial for distinguishing between Grades I, II and III.

### The change in the talofibular distance on the affected side with respect to the number of days

We found that the predicted time for the change in the talofibular distance on the affected side to match the median value for the unaffected side was 14.5, 43.2 and 45.6 days for Grade I, II and III injuries, respectively. No significant difference was observed between Grades II and III regarding the regression slopes. A priori power analysis indicated that our sample size was sufficient for detecting a moderate effect size (more than 55 participants) but not for a small effect size (395 participants needed), necessitating caution when interpreting results for Grades II and III. The recovery of anterior ankle displacement is typically achieved within 6 weeks after ligament rupture, aligning with previous basic research [19]. The ligament healing process occurred through overlapping phases of inflammation, repair, and remodelling, with remodelling occurring around 6 weeks after injury [2, 17, 19]. Therefore, recovery for anterior ankle joint displacement was 6 weeks regardless of partial or complete ligament tears. Croy et al. [4] reported slight improvement in the change in the talofibular distance 6 weeks after LAS, and Hubbard and Cordova [8] noted persistent displacement 8 weeks after LAS. However, their studies involved university‐recruited participants with unknown injury timelines and frequencies. Hubbard and Hicks‐Little [9], in their systematic review, indicated that joint stability typically recovers within 6 weeks to 3 months following LAS; however, the review did not include US‐guided stress testing, which may have led to varied severity diagnoses. In our study, the initial severity was diagnosed using US, and distinct disease courses were identified based on the severity. However, the relationship between the recovery of joint displacement and outcomes such as function, RTS, and the recurrence rate of injury was not clarified. Karlsson et al. [13] reported positive outcomes with conservative treatment protocols for grades II and III. Accurate initial diagnosis is crucial for appropriate treatment planning. Although current ankle sprain guidelines [25] recommend uniform durations for external fixation and brace use regardless of severity, future policies should consider severity‐specific treatment.

### Limitations

This study had a few limitations. First, several uncontrollable confounding factors, including swelling and pain on the body surface, as well as generalised ligamentous laxity, were not assessed. Additionally, different rehabilitation protocols for Grade I to III injuries may have influenced the outcomes related to joint displacement. As many patients develop these symptoms after LAS, future studies should investigate their relationship with anterior ankle joint displacement. Second, the median age of participants was 16.0 years, with a maximum age of 54 years, and 82% (83/101) were student athletes. This younger adolescent athlete population contrasts with that in the study by Doherty et al. [5], where the median age was 22.8 years. Third, there might have been participant selection bias. The clinic requires regular follow‐up examinations based on case severity; however, 12 (12%) out of 101 cases did not undergo follow‐up. This suggests that the study may have included only those with good compliance. However, the comparison between groups for Grades I and III was inconclusive because of insufficient power. Finally, although the use of a single experienced examiner ensured intra‐examiner reliability in this study, the potential for variability in results because of examiner skill raises concerns about generalisability. While the placement of the ROI demonstrated high reproducibility with an ICC of 0.91 in this study, variability in ROI placement could arise if the high‐echoic area expands owing to ligament scarring or other factors. Despite these limitations, this study evaluated a relatively young cohort for injury severity using US imaging to measure joint displacement, with follow‐up generating 276 data points, thereby providing significant value in the context of US imaging.

## CONCLUSION

The estimated time to recover ultrasonographic anterior ankle joint displacement in patients with initial acute LAS was different between LAS Grades I and II–III, but there was no difference between grades II and III. Evaluating the presence and severity of ligament rupture using US is crucial for estimating the time required to resolve the difference between the unaffected and affected sides.

## AUTHOR CONTRIBUTIONS

*Conceptualization*: Yuto Uchida, Masashi Kawabata, Kazuya Takagi, and Kazuma Miyatake. *Methodology*: Yuto Uchida, Masashi Kawabata, and Hiroyuki Watanabe. *Formal analysis*: Yuto Uchida and Masashi Kawabata. *Investigation*: Yusuke Kumazawa, Yuto Uchida, and Masashi Kawabata. *Resources*: Yusuke Kumazawa. *Data curation*: Yusuke Kumazawa, Yuto Uchida, and Masashi Kawabata. *Writing—original draft preparation*: Yuto Uchida. *Writing—review and editing*: Masashi Kawabata, Takumi Kobayashi, and Tomonori Kenmoku. *Supervision*: Hiroyuki Watanabe and Naonobu Takahira. *Project administration*: Masashi Kawabata and Naonobu Takahira. All authors have read and agreed to the published version of the manuscript.

## CONFLICT OF INTEREST STATEMENT

We have a joint research agreement with Konica Minolta, Inc. No research funding has been received from Konica Minolta, Inc. Kazuma Miyatake, Masashi Kawabata, and Yusuke Kumazawa have received speaker honoraria from Konica Minolta, Inc. Additionally, Kazuma Miyatake has received equipment support (ultrasound diagnostic device) from Konica Minolta, Inc.

## ETHICS STATEMENT

This study was conducted in accordance with the Declaration of Helsinki and was approved by the Ethics Committee of Kitasato University (study number 2021‐025‐2). The requirement for informed consent was waived by this committee before the start of this retrospective study, given that only anonymized clinical data were used for the analyses conducted in the study.

## Data Availability

The data sets generated and analysed during the current study are available from the corresponding author upon reasonable request.
